# Detectable Vesicular Stomatitis Virus (VSV)–Specific Humoral and Cellular Immune Responses Following VSV–Ebola Virus Vaccination in Humans

**DOI:** 10.1093/infdis/jiy565

**Published:** 2018-11-17

**Authors:** Joseph H Poetsch, Christine Dahlke, Madeleine E Zinser, Rahel Kasonta, Sebastian Lunemann, Anne Rechtien, My L Ly, Hans C Stubbe, Verena Krähling, Nadine Biedenkopf, Markus Eickmann, Sarah K Fehling, Flaminia Olearo, Thomas Strecker, Piyush Sharma, Karl S Lang, Ansgar W Lohse, Stefan Schmiedel, Stephan Becker, VSV-Ebola Consortium (VEBCON), Claire-Anne Siegrist, Angela Huttner, Marylyn M Addo, Stephan Becker, Verena Krähling, Phillip Bejon, Patricia Njuguna, Francis Ndungu, Peter G Kremsner, Jessica S Brosnahan, Selidji Todagbe Agnandji, Sanjeev Krishna, Marie Paule Kieny, Kayvon Modjarrad, Vasee Moorthy, Patricia Fast, Barbara Savarese, Olivier Lapujade, Marylyn M Addo

**Affiliations:** 1 First Department of Medicine, University Medical Center Hamburg-Eppendorf, Hamburg; 2 German Center for Infection Research, partner site Hamburg-Lübeck-Borstel-Riems; 3 Division of Infectious Diseases, University Medical Center Hamburg-Eppendorf; 4 Leibniz Institute for Experimental Virology, Heinrich Pette Institute, Hamburg; 5 Division of Infectious Diseases, Department of Medicine II, Ludwig Maximilian University, Munich; 6 Institute for Virology, Philipps University Marburg; 7 German Center for Infection Research, partner site Gießen-Marburg-Langen; 8 Institute of Immunology, University Duisburg-Essen, Essen, Germany

**Keywords:** vesicular stomatitis, virus, VSV-EBOV, vaccine, Ebola virus, vector immunity, preexisting immunity

## Abstract

In response to the Ebola virus (EBOV) crisis of 2013–2016, a recombinant vesicular stomatitis virus (VSV)–based EBOV vaccine was clinically tested (NCT02283099). A single-dose regimen of VSV-EBOV revealed a safe and immunogenic profile and demonstrated clinical efficacy. While EBOV-specific immune responses to this candidate vaccine have previously been investigated, limited human data on immunity to the VSV vector are available. Within the scope of a phase 1 study, we performed a comprehensive longitudinal analysis of adaptive immune responses to internal VSV proteins following VSV-EBOV immunization. While no preexisting immunity to the vector was observed, more than one-third of subjects developed VSV-specific cytotoxic T-lymphocyte responses and antibodies.

## Teaser

This study investigates preexisting and vaccine-induced vector immunity in 30 participants of a Phase-1 VSV-EBOV Ebola vaccine trial. No preexisting immunity was detected, however humoral and cell-mediated immunity against internal VSV proteins was observed in up to 36% of vaccines.

Between 2013 and 2016, Africa experienced the largest epidemic of Ebola virus disease (EVD) in history. The unprecedented spread of EVD led to acceleration of vaccine trials. In late 2014, the Ebola virus (EBOV) vaccine candidate VSV-EBOV (V920) entered phase 1 clinical testing [1, 2].

VSV-EBOV is based on a recombinant live-attenuated vesicular stomatitis virus (VSV) expressing the EBOV glycoprotein (GP) instead of the VSV wild-type (VSVwt) glycoprotein G [3]. Preclinical studies revealed fast induction of VSV-EBOV–specific immunity and high efficacy in single-dose vaccine regimens, hereby suggesting its suitability, particularly for outbreak scenarios [4]. The first human efficacy data for the VSV platform were generated by testing VSV-EBOV in a clinical phase 2/3 trial in Guinea with evidence for rapid and robust protection from EVD [5]. In contrast to comprehensive analyses on vaccine-induced EBOV-specific immune responses, vector immunity to VSV in humans has not been investigated to date.

Preexisting immunity represents a potential drawback for viral-vector vaccines with a possible risk for reduced vaccine efficacy. This has previously been reported, for example, for adenovirus and human parainfluenza virus 3 (HPIV-3) platforms [6, 7]. While the potential clinical impact of preexisting immunity of the adenovirus type 5–based EBOV vaccine has been described [6], scarce data are available for the VSV platform.

We performed a comprehensive investigation of preexisting and induced vector immunity against VSV in 30 healthy subjects immunized with 3 different doses (3 × 10^5^ plaque-forming units [PFU], 3 × 10^6^ PFU, and 2 × 10^7^ PFU) [1, 8]. Preexisting immunity to the vector was not detectable, but study subjects generated VSV-specific immune responses as measured by enzyme-linked immunosorbent assay (ELISA), enzyme-linked immunospot assay (ELISpot), and flow cytometry.

Overall, up to 36% of vaccinees generated vector-directed immune responses. However, the magnitude of responses was highly variable between trial participants. Considering the expanding use of VSV vaccine vectors in emergency vaccine efforts, such as in the recent EVD outbreaks in the Democratic Republic of the Congo (DRC) [9], the investigation of vector-directed immunity requires urgent attention. The data presented here provide a first insight into VSV vector immunity in the context of human immunization with VSV-EBOV and may add value to strategic vaccine design efforts.

## METHODS

### Study Design and Participants

NCT02283099 was a phase 1 trial of escalating doses of VSV-EBOV in healthy adults. Details about the trial and study protocol have been reported previously [1, 8].

### Humoral Immunity to VSV and EBOV-GP

Plasma was analyzed for the presence of VSV matrix–specific (VSV-M) antibodies using the Recombivirus Human Anti-VSV Indiana M Protein ELISA Kit (Alpha Diagnostic). ELISA to EBOV-GP was performed as previously described [1]. VSV neutralization assays were conducted with plasma and incubated with 500 PFU of VSVwt (Indiana) on Vero cells. Plasma from VSVwt-infected C57BL/6 mice served as positive control. Neutralization assays against EBOV particles were performed as previously described [1].

### VSV-Specific T-Cell Responses

VSV-specific T cells were analyzed using cryopreserved peripheral blood mononuclear cells (PBMCs) and overlapping peptide pools (OLPs) spanning the VSV nucleoprotein (VSV-N) (Supplementary Table 1). Following overnight resting, PBMCs were incubated for 6 hours and 16 hours at 37°C with VSV-N OLPs or with ultraviolet-inactivated VSVwt, respectively, in the presence of CD28/CD49d and GolgiPlug/GolgiStop (BD Bioscience). Negative controls were treated with R10 supplemented with dimethyl sulfoxide, or R10 alone. Phorbol 12-myristate 13-acetate (PMA)/ionomycin and CEF (cytomegalovirus [CMV]/Epstein-Barr virus/influenza peptides) served as positive controls. We analyzed expression of tumor necrosis factor alpha (TNF-α), interleukin 2, interferon gamma (IFN-γ), and CD107a. Cells were acquired on a LSRFortessa (BD Bioscience) and analyzed using FlowJo10 software. The gating strategy is shown in Supplementary Figure 2.

### Statistical Analysis

Statistical analysis was performed using GraphPad Prism software (version 7.02.). Intergroup differences were analyzed using Mann–Whitney–Wilcoxon test.

## RESULTS

### Humoral Immune Responses to VSV

**Figure 1.**
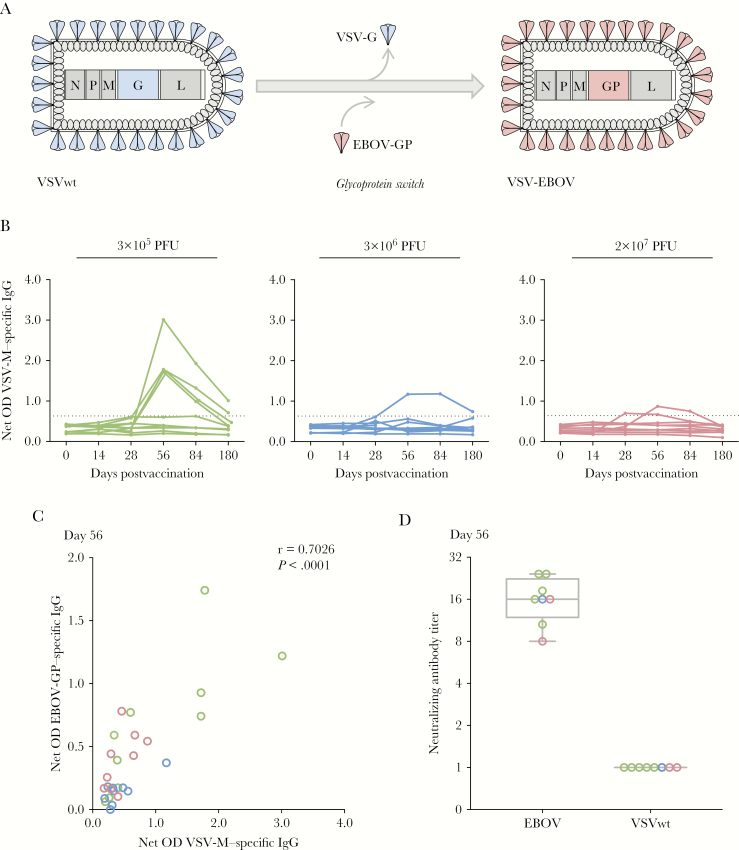
Humoral responses against vesicular stomatitis virus (VSV). *A*, Structure and design of VSV–Ebola virus (EBOV) vaccine. VSV glycoprotein G (G) is replaced by EBOV glycoprotein (GP), while nucleoprotein (N), phosphoprotein (P), matrix protein (M), and RNA-dependent RNA polymerase (L) correspond to the VSV backbone vector. *B*, VSV-M–specific antibodies were generated following VSV-EBOV immunization in humans. VSV-M antibody titers were assessed by enzyme-linked immunosorbent assay at baseline and days 14, 28, 56, 84, and 180 postvaccination. Results are expressed as corrected optical density (OD) values. The dashed line depicts the threshold for a positive antibody response, calculated as the median on day 0 of all subjects ± 3 standard deviations. VSV-M–specific antibodies are detectable in 8 subjects (3 × 10^5^ plaque-forming units [PFU], 5 of 10 subjects; 3 × 10^6^ PFU, 1 of 10 subjects; 2 × 10^7^ PFU, 2 of 9 subjects). *C*, Positive correlation between OD values of VSV-M and EBOV-GP–specific immunoglobulin G (IgG) at day 56 postvaccination. *D*, VSV-M–positive subjects were analyzed for generation of neutralizing antibodies against VSV wild-type (VSVwt; (n = 8). Neutralizing antibodies against infectious EBOV isolate Mayinga but not against VSV-M were detected. Statistical analysis was performed with Mann–Whitney–Wilcoxon test.

Given the very recent introduction of VSV vectors to human study populations [1, 5, 10], there is still a need to fill critical knowledge gaps related to the vector’s use in humans. While immune responses to several vaccine vector inserts have been studied in detail [8, 10], data on immunity to vector-specific proteins (Figure 1A) are scarce.

In the current study, we focused on adaptive immunity against VSV-M and VSV-N. First, vector-specific antibody responses against VSV-M were analyzed by ELISA using longitudinal collected plasma samples. None of the subjects showed preexisting VSV-M–specific antibodies (day 0) (Figure 1B); however, 28% of the vaccinees (8/29) developed transient VSV-M–specific antibodies, peaking on day 56 postvaccination. We observed VSV-M–specific antibodies in 5 of 10 subjects of the 3 × 10^5^ PFU cohort, in 1 of 10 subjects of the 3 × 10^6^ PFU cohort, and in 2 of 9 subjects of the 2 × 10^7^ PFU cohort. The low-dose group showed the highest magnitude of antibody titers.

We next sought to investigate the association of vector- and EBOV-specific antibody responses. The induction of EBOV-GP antibodies in vaccinees has previously been described [1, 8]. A correlation of VSV-M with EBOV-GP antibody responses using optical density values from day 56 (Figure 1C) revealed a strong positive correlation (*r* = 0.7; *P* < .0001), indicating that vaccine responders generated antibodies not exclusively to the target antigen EBOV-GP, but also to the viral vector itself.

Subsequently, the function of VSV-specific antibodies was further analyzed by evaluating the capacity to inhibit VSVwt replication. We analyzed a subset of vaccinees based on their generation of VSV-M–specific antibodies (Figure 1B). While incubation of plasma with VSVwt showed no inhibition of viral replication, the capacity to neutralize EBOV particles was detected in all subjects (Figure 1D) [1, 8].

### T-Cell–Mediated Immune Responses to VSV

**Figure 2.**
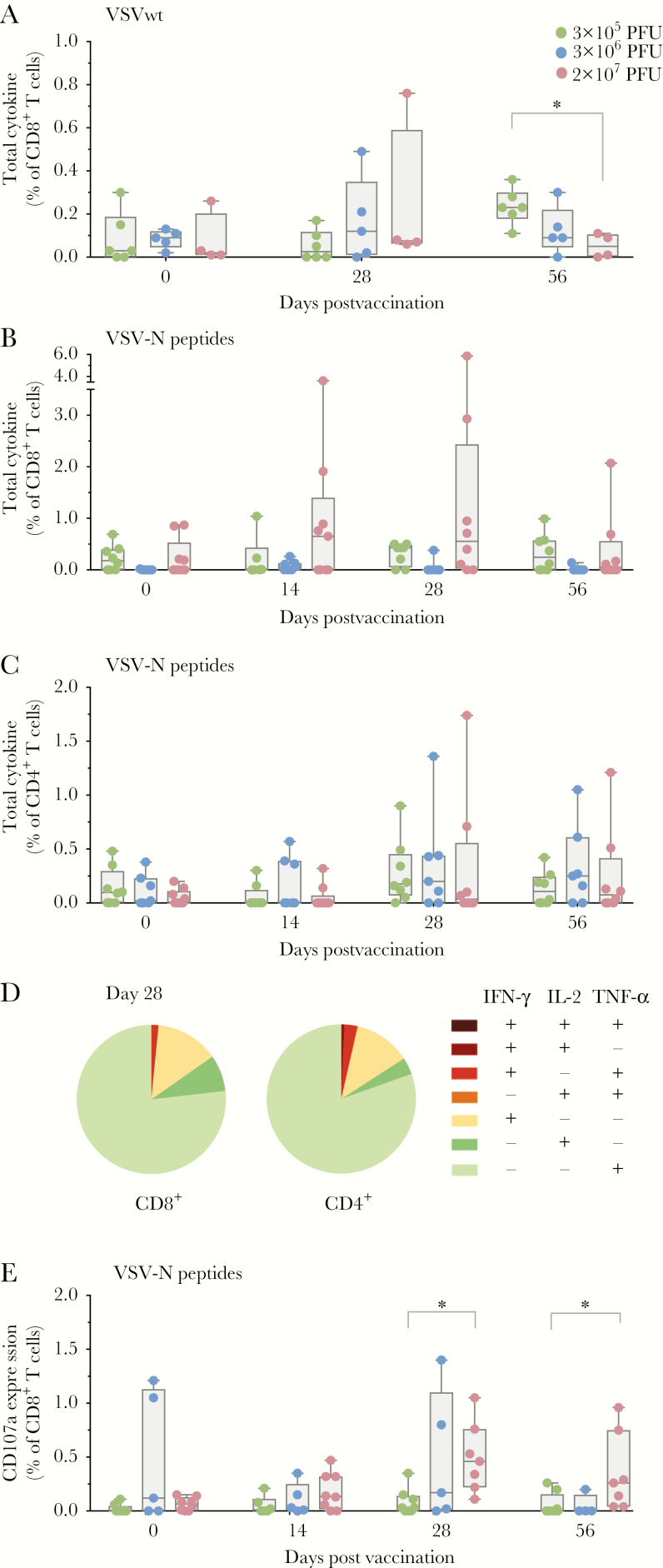
Antigen-specific T cells against vesicular stomatitis virus (VSV). *A*, VSV wild-type (VSVwt)–specific T-cell responses. Peripheral blood mononuclear cells were stimulated with ultraviolet-inactivated VSVwt. Graph depicts the observed interferon gamma (IFN-γ)/interleukin 2 (IL-2)/tumor necrosis factor alpha (TNF-α) secretion of CD8^+^ T cells measured by flow cytometry. Each dot represents summarized cytokine responses of CD8^+^ T cells for 1 subject (3 × 10^5^ plaque-forming units [PFU]: n = 6; 3 × 10^6^ PFU: n = 5; 2 × 10^7^ PFU: n = 4). A significant intergroup difference between the low- and high-dose group was observed on day 56 (Mann–Whitney test, *P* = .01). *B* and *C*, Cytokine responses of CD8^+^ and CD4^+^ T cells (IFN-γ/IL-2/TNF-α) following stimulation with VSV nucleoprotein (VSV-N) overlapping peptide pools, respectively. Cytokine secretion was measured by flow cytometry (3 × 10^5^ PFU: n = 8; 3 × 10^6^ PFU: n = 7; 2 × 10^7^ PFU: n = 9). *D*, Pie charts represent the functionality of specific T cells to VSV-N peptide pools at day 28 following immunization. Shown are the proportions of VSV-N–specific memory CD8^+^ (left) and CD4^+^ (right) cells that produce any combinations of the 3 measured cytokines. Pie charts represent the mean value of 9 subjects from the high-dose cohort. *E*, Cytotoxic T-lymphocyte (CTL) response following stimulation with VSV-N peptides. Flow cytometry analysis of the degranulation marker CD107a in the CD8^+^ T-cell subset (3 × 10^5^ PFU: n = 10; 3 × 10^6^ PFU: n = 5; 2 × 10^7^ PFU: n = 8). The magnitude of CTL responses revealed significant intergroup differences on days 28 and 56 (Mann–Whitney–Wilcoxon test, *P* = .0045 and *P* = .0095, respectively). Comparing T-cell responses following VSV-N peptide stimulation revealed an increased response to VSV-N peptides in 3 vaccinees, showing induced cytokine or CD107a expression in CD8^+^ or CD4^+^ T cells. Box and whiskers show minimum to maximum; line shows the median. Statistical analysis was performed with Mann–Whitney–Wilcoxon test (*P < .05). Green: 3 × 10^5^ PFU; blue: 3 × 10^6^ PFU; red: 2 × 10^7^ PFU.

T-cell responses against the vector may eliminate VSV-infected cells and thereby modulate vaccine efficacy. We first evaluated T-cell responses against the whole VSVwt particle (Figure 2A). Total cytokine responses of CD8^+^ T cells stimulated with VSVwt were detectable, but were of low magnitude. While the 2 higher-dose cohorts revealed a peak of cytokine-producing CD8^+^ T cells at day 28, the low-dose group showed an increase of total cytokine responses at day 56.

We next analyzed T-cell responses following stimulation of PBMCs with OLP pools covering VSV-N (Figure 2B–E). Similar to stimulation with VSVwt particles, the high-dose cohort showed increased responses peaking at day 28 compared with the lower-dose cohorts. We identified a predominance of VSV-N–specific CD8^+^ (Figure 2B) over CD4^+^ T cells (Figure 2C). The analysis of polyfunctionality using Boolean gating predominantly revealed VSV-N–specific monofunctional T cells expressing TNF-α (Figure 2D). A smaller subset of CD4^+^ and CD8^+^ T cells expressed IFN-γ. Furthermore, we observed a minor expansion of dual-functional CD8^+^ T cells (TNF-α^+^IFN-γ^+^). Note that the induction of IFN-γ expression following VSV-N stimulation was validated in a subset using ELISpot (Supplementary Figure 1).

Next, cytotoxic T-lymphocyte (CTL) responses were investigated by CD107a staining. After stimulation with VSV-N OLPs, 75% of subjects of the high-dose cohort showed at least a 2-fold induction of CD107a expression in CD8^+^ T cells at day 28 (Figure 2E).

Taken together, no preexisting humoral or cell-mediated VSV-specific immune responses were detected in this German study population. However, one-third of vaccinees developed nonneutralizing antibodies and T-cell responses against VSV proteins following immunization with VSV-EBOV.

## DISCUSSION

VSV-EBOV represents a promising vaccine candidate and has only recently entered human clinical trials and is now administered in compassionate use programs. The VSV platform is currently being considered for immunization strategies for several World Health Organization priority diseases (http://www.who.int/blueprint/en/).

In the context of viral-vector vaccines, vector immunity potentially represents an obstacle for vaccine efficacy. Through the successful development of VSV-based vaccines against highly pathogenic viruses with geographically overlapping endemic areas (eg, Africa), the effect of preexisting immunity to the vaccine vector requires careful attention. Given the lack of data on VSV-directed immune responses in humans, we here addressed if natural immunity against VSV is detectable and if VSV-EBOV elicits adaptive immunity against VSV proteins following immunization.

In the study population, no preexisting immune responses were detected, possibly related to the fact that all vaccinees originated from and reside in Europe. VSV is endemic in North, Central, and South America, and generally infects cattle. Humans with a high risk of VSVwt exposure are individuals living in these regions in close contact to livestock [3].

While preexisting immunity may be a minor problem for current vaccine trials, acquired vector immunity could emerge as a relevant factor given the increasing number of clinical vaccine trials applying the VSV platform (eg, Partnership for Research on Ebola Virus in Liberia [PREVAIL], Sierra Leone Trial to Introduce a Vaccine Against Ebola [STRIVE]) [2]. In the context of the recent EVD outbreaks in DRC, the number of humans immunized with VSV-EBOV is further increasing [9].

The effect of acquired preexisting immune responses against a viral vaccine vector has been described in several studies [6, 7]. Possible explanations for this phenomenon include humoral or cellular immunity mediated against vector proteins. For example, the impact of cell-mediated immune responses against a vector nucleoprotein has been shown in mice vaccinated with vaccinia virus [11]. The authors demonstrated stronger T-cell responses against the vector nucleoprotein compared to inserted foreign epitopes, suggesting elimination of vaccine-infected cells upon second encounter. Given the observed induction of CTL responses to VSV-N in 36% of vaccinees in our study, there is the potential for vector-directed immune responses to restrict VSV-EBOV replication, which has to be further explored in future studies.

Beyond VSV-N–specific T cells, vector-directed antibodies may also be implicated in reducing vaccine efficacy. In this context, one phase 1 study using homologous prime-boost administration with VSV-EBOV failed to demonstrate efficient antibody induction and showed no significantly increased neutralization titers following a homologous boost on day 28 [12], possibly suggesting that decreased vaccine efficacy may have been modulated by induced vector-directed responses, as discussed by the authors.

While the exact impact of induced vector-directed antibodies remains unclear to date, the transiently detected binding VSV-M antibodies potentially contribute to dampening of vaccine responses upon second encounter and may need to be taken into consideration for future prime-boost implementations or further VSV-associated vaccinations. However, it is noteworthy that all tested samples in this study neutralized EBOV and none VSVwt; therefore, the neutralization capacity seems to be specific to and mediated by EBOV-GP [1, 8] (Figure 1D).

While concern for an impact of vector-directed antibodies on vaccine efficacy remains, a recent study in nonhuman primates assessed the effect of vaccine vector–induced preexisting immunity to VSV in a preclinical study using Lassa virus (LASV) and EBOV vaccine constructs (boost: day 90) [13]. Cynomolgus macaques vaccinated with VSV-LASV and challenged with LASV were vaccinated with VSV-EBOV 3 months later, and subsequently challenged with a lethal dose of EBOV. Despite high VSV-specific antibody titers at the time of VSV-EBOV immunization, the animals were completely protected from lethal EBOV challenge.

There may also be options to minimize vector immunity. One strategy might be the optimization of prime-boost intervals. One volunteer immunized with VSV-EBOV received a homologous boost 6 months later. Here, a strong boosting effect on antibody titers was demonstrated (C. A. Siegrist, personal communication). Alternatively, genetic modification of the viral vector, as shown for the HPIV-3-platform, may also be explored in this context [7].

We observed distinct magnitudes of humoral responses among participants immunized with different vaccine doses. Interestingly, low-dose subjects showed the greatest number of assay responders and the highest antibody titers. This may be related to the fact that the low number of virus particles administered to subjects immunized with the low dose potentially induces weaker innate immune responses, protracting viral replication compared to subjects, who received higher doses. Therefore, the vaccine may replicate for prolonged periods, resulting in stronger adaptive immune responses, as previously described for the live 17D yellow fever vaccine [14]. Another potential explanation may be related to CMV seropositivity, as this has been described as a potentially beneficial factor for antivaccine responses following influenza immunization [15]. The low-dose cohort in the present study demonstrated a higher number of CMV-positive subjects (n = 4) in contrast to only 1 CMV-seropositive participant in the middle-dose and high-dose cohorts, respectively (data not shown). A correlation analysis of CMV against VSV and EBOV titers in the low-dose cohort demonstrated a strong linear relationship (Supplementary Figure 3).

Our study detected vector-specific immune responses in more than one-third of subjects following VSV-EBOV immunization and emphasizes the need to explore vector immunity as part of vaccine evaluations with this new vaccine vector. Homologous boosting may be less effective due to vector-directed antibody responses. Furthermore, cell-mediated responses to internal VSV proteins might inhibit efficient vector replication by limiting virus spread. As humoral and cell-mediated responses peaked at days 28 and 56, VSV-based vector immunity could potentially be minimized by readministration of the vaccine at later time points.

In conclusion, our results highlight that immune responses against VSV are elicited after a single vaccine administration of VSV-EBOV in human subjects. Future implementations using the VSV platform require careful consideration of vector immunity with respect to prime-boost or other immunization strategies.

## Supplementary Data

Supplementary materials are available at *The Journal of Infectious Diseases* online. Consisting of data provided by the authors to benefit the reader, the posted materials are not copyedited and are the sole responsibility of the authors, so questions or comments should be addressed to the corresponding author.

## Supplementary Material

Supplementary Table 1

Supplementary Figures
