# Supplementary material for: Detectable Vesicular Stomatitis Virus (VSV)–Specific Humoral and Cellular Immune Responses Following VSV–Ebola Virus Vaccination in Humans
Source: J Infect Dis. 2018 Nov 17;219(4):556–61. doi: 10.1093/infdis/jiy565 (PMC6350948; doi:10.1093/infdis/jiy565)
Supplement: Supplementary Table 1 [file jiy565_suppl_supplemental_table_1.docx]

**Supplemental Table 1**


VSV-N OLPs, 15-mers with 11-amino acid overlap (*peptides and elephants*)
