# Supplementary figures and images for: Detectable Vesicular Stomatitis Virus (VSV)–Specific Humoral and Cellular Immune Responses Following VSV–Ebola Virus Vaccination in Humans
Source: J Infect Dis. 2018 Nov 17;219(4):556–61. doi: 10.1093/infdis/jiy565 (PMC6350948; doi:10.1093/infdis/jiy565)

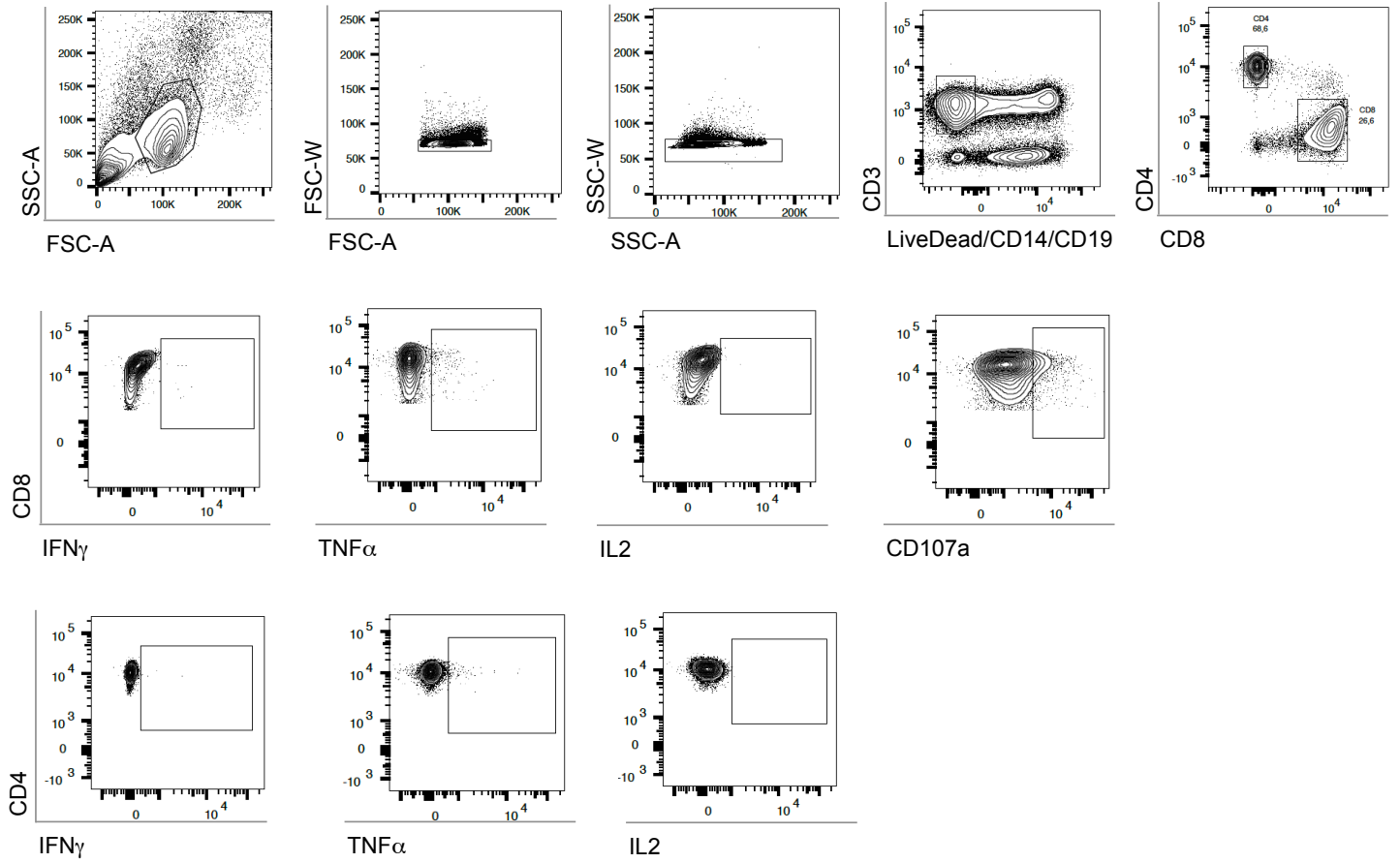

day 56

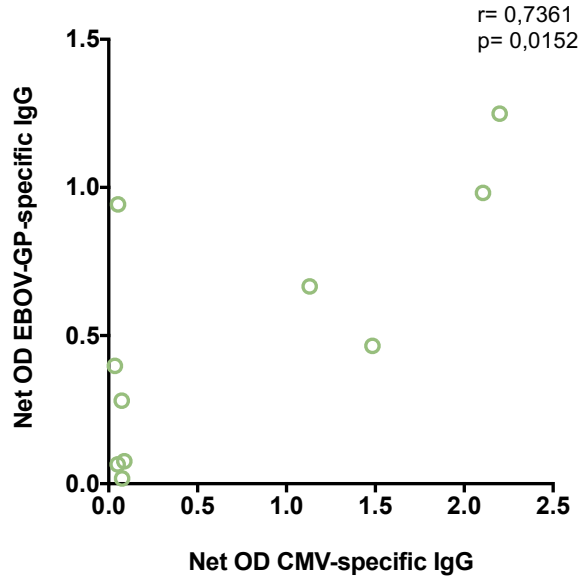

day 56

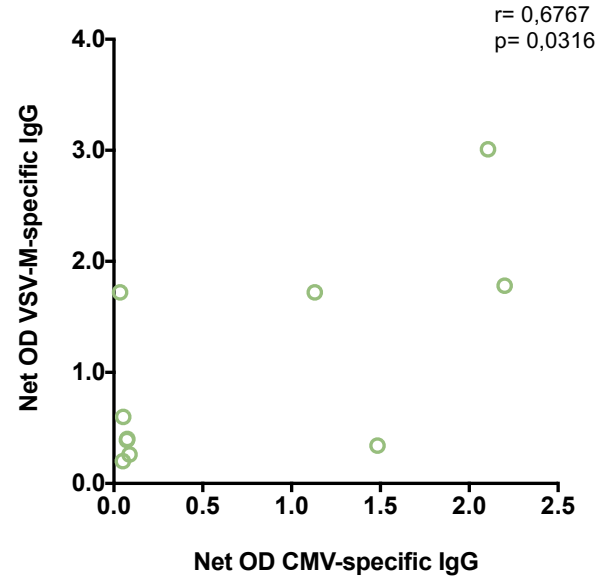

Supplement: Supplementary Figures [file jiy565_suppl_supplemental_figures.pdf]
